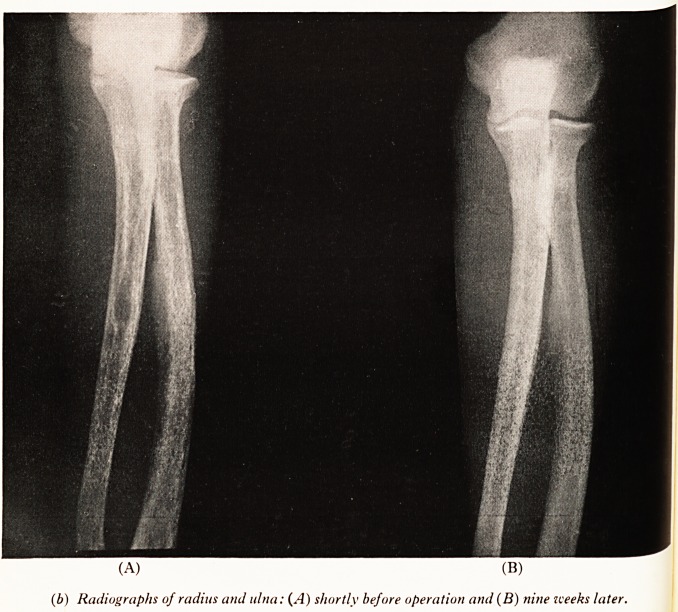# Generalized Osteitis Fibrosa with Parathyroid Adenoma

**Published:** 1956-07

**Authors:** T. F. Hewer


### GENERALIZED OSTEITIS FIBROSA WITH PARATHYROID ADENOMA

(*A Clinical Pathological Conference of the University of Bristol Medical School.*)

CHAIRMAN: PROFESSOR T. F. HEWER

*Dr. J. M. Naish:* I first saw this patient in Snowdon Road Hospital. As she was only forty-five and therefore much younger than is usual for patients in this hospital I thought as I approached the bed that she had some interesting neurological condition or disseminated sclerosis but I was told that she had had some pain around the groin, that her doctor had sent her for X-ray of the pelvis, and that a large destructive lesion had been found in one of the pubic rami. A surgeon saw her and advised that she should be admitted to a chronic hospital: the diagnosis was secondary tumours of bone. When I first looked at this X-ray picture of the pubis and one of the skull, which was taken because she had exophthalmos of one eye, I fell for the diagnosis and looked for a primary, but I was struck by her good general condition. There was no obvious primary; the thyroid and breasts were normal. The uterus had been removed some years previously. The kidneys were just palpable—we had a pyelogram done and that was normal. I felt it was probably not a malignant condition. When she could walk properly we let her go home and I saw her in out-patients' on two occasions and then readmitted her to Southmead. At that time the condition which was running through my mind was myelomatosis or xanthomatosis: I suggested that a biopsy should be done and Dr. Brown had the specimen. He gave a most interesting report, as a result of which we had the serum calcium and phosphate levels estimated and they were grossly abnormal: serum alkaline phosphatase 58 units per 100 ml., serum inorganic phosphate 2·5 mg. per cent., serum calcium 14·4 mg. per cent. These were so abnormal that they entirely confirmed Dr. Brown's suggestion and as X-ray pictures were also confirmatory we sent her to the surgeon to find the tumour which we could not feel!

*Mr. A. G. McPherson:* My job was a straightforward one; I had to look for a parathyroid tumour. She was pretty thin; it was a scraggy neck and there was nothing to feel in it. I did a standard thyroid incision and exposed the whole thyroid by dividing across the strap muscles. I started on the left lobe, looked at all aspects, divided both main arteries and finding nothing I divided the pre-vertebral fascia and found nothing except one or two little nodules on the thyroid. I went to the right side and again I turned the lobe practically over, examining the whole of the back and could feel nothing. I then divided the fascia over the pre-vertebral muscles, slipped my finger down there and then I felt something; it was in front of my finger, and after that it was relatively easy to dissect it out. (Two photographs taken during the operation were then shown.)

*Dr. N. J. Brown:* Let us first consider the bone biopsy. The histological changes are well illustrated by the slides. (See Plate IX (*a*).) The principal features are erosion of the bone and replacement by fibrous tissue. In places (Plate IX (*b*)) there are clusters of osteoclasts nibbling away the bone. There is very little evidence of cyst formation. It is clearly a form of fibrous dysplasia of bone and Dr. Lewis and I both felt that there was a strong possibility of this being a case of generalized osteitis fibrosa cystica, due to overactivity of the parathyroids. We sent out a report to this effect and suggested further investigation along these lines.

Now to turn to the parathyroid tumour which Mr. McPherson removed. This measured 4×2×2 cm. and was mainly solid with a few small cysts. Histologically (Plate X (*a*)) it shows a uniform picture, being composed of small cells with regular round nuclei. It is a simple parathyroid adenoma composed of "chief" cells.

*Dr. G. R. Airth:* My first contact with this patient was a request from her family doctor for examination of the right hip joint; a chest film had been taken some days

(*a*) *Biopsy of pubis: erosion of bone and replacement by fibrous tissue.*

(*b*) *Biopsy of pubis: osteoclastic resorption of bone.*

PLATE X

(*a*) *Parathyroid adenoma; photomicrograph of operation specimen.*

(A) (B)

(*b*) *Radiographs of radius and ulna:* (*A*) *shortly before operation and* (*B*) *nine weeks later.*

previously. An extensive lesion of the symphysis pubis was present and others involved the right acetabular floor and the left ischial ramus. I received vague information from the patient that she had had "half her inside out" and was guilty of calling the bone lesions metastases on these thin premises. I advised reference to a physician. Later I learned that she had also been seen at the Bristol General Hospital, where similar conclusions were drawn. Six weeks later I saw films of skull and ribs; the multiple lesions still suggested metastases. In the search for a primary lesion producing osteolytic metastases, pyelograms showed no evidence of a renal lesion. The patient appeared again, nearly seven months after the hip episode, and it was clear that in spite of new lesions, and enlargement of the old, the cortex was being pushed aside and not being destroyed; myelomatosis was suggested. Finally, when biopsy and biochemical findings were known, the most appropriate bones were examined for the first time; the radius (Plate IX (*b*)) showed severe osteoporosis, with absorption of compact bone and a cystic space in its middle third. The metacarpals and phalangeal shafts showed many small cysts. Spontaneous fractures were shown where the cystic spaces occupied much of the bone thickness, as in the case of the ribs and ischium.

*Dr. Naish:* Ten days after the operation she fractured her femur—apart from that she had an uneventful recovery.

*Dr. Airth:* Nine weeks after the removal of the parathyroid tumour the healing process was already well advanced (Plate X (*b*)); the skull looked entirely normal, the pubic lesion was shrunken and sclerosed, the cysts had entirely disappeared, and in the case of the radius the only abnormality was a small midshaft area of sclerosis.

*Dr. F. J. W. Lewis:* The laboratory findings confirmed the diagnosis. There was a very high alkaline phosphatase, a raised plasma calcium and lowered inorganic phosphate. Hyperparathyroidism causes increased excretion of phosphate and therefore lowers the serum phosphate throwing out the calcium phosphate balance and thus causing a rise in calcium. On the findings obtained I felt quite confident of our diagnosis.

*Dr. J. E. Cates:* Many parathyroid tumours are not associated with decalcification; this has been noticed particularly in the North American continent where the dietary intake of calcium is high. In these cases the phosphatase may be quite normal, but the serum calcium is high and quite often there are renal stones. Another interesting feature of the raised calcium level is the mental changes; the patients become depressed and mentally slow. It would also be interesting to know whether this patient had any signs of tetany when the tumour was taken out.

*Dr. Naish:* This is a very rare condition and the only case I have seen myself. With regard to the renal complications; they can be very serious. I do not know anything about the patient's diet—I think it was quite normal. About her mental changes: she was an exceptionally nice person and I think she probably felt very depressed and miserable but she never showed unpleasant traits or behaviour. About getting post-operative tetany: we tried to guard against this by giving her additional calcium four days before the operation and two or three weeks after. We had no post-operative complications.

*Mr. R. V. Cooke:* I can speak of quite a few cases: one of the two most interesting and instructive ones was one from Southmead Hospital about twenty years ago: there was in Southmead a sort of dwarf—a little boy who had had many stones removed from his kidney and he had a very high blood calcium—18·8—and we found this parathyroid tumour. The strange thing was that he had been regarded as a mental case—almost an idiot—and rather the joke of the hospital. He had the tumour removed about the first week in December and by Christmas he was the wag of the ward! He became bright and intelligent and good fun: quite a different picture.

The next case was one which was missed and I suppose every excuse was found for missing it! This was a patient who was in the General Hospital about fifteen years ago with a large swelling in her face; she had a tumour removed and it was a big clean cavity when I saw it. Fortunately for her, just before she left hospital, she developed a swelling in the upper end of her tibia which was visible and the surgeon asked me to see her. I saw her and the hole in her cheek and the lump in the tibia and I said to the students "Now what is the diagnosis?" They said it was very hard and I said that there were many suggestions we might make. They thought secondary malignant metastases from this tumour in the upper jaw. She was a young woman and she was pretty fit physically but dull mentally. I said, "I think this is a parathyroid tumour and what is more, here it is!" When she swallowed, you could feel this tumour coming out of the superior thoracic border and it was quite easy to feel. So here was the whole clinical picture—no need for any tests. It was so unusual that Professor Perry, to whom I referred the case, thought it was a figment of my imagination but I am not an imaginative person and I said, "Well—there it is." We took pictures of other bones, the long bones, and there were many cystic spaces. She was removed from the surgical ward and fractured her femur on the way to the medical ward. We operated on her: I made a tiny little incision about two inches long and removed the tumour: that was the whole operation and it took about a quarter of an hour. She got well and remained well for a number of years. After about seven years, during which time her blood chemistry was reviewed from time to time in the out-patients' department, things started to go wrong again. Her calcium went up and the condition was recurring: it was not a persistent case but a recurring case. She began to get cysts again and she fractured another long bone. Her mental condition became quite definitely better after the first operation; she became quite charming. When we explored her the second time, I examined the thyroid thoroughly and found nothing very dramatic in the way of a parathyroid tumour. I did eventually find a small nodule about 1 cm. in diameter which was different in colour from the rest of the thyroid, then I found another and another—there were three. Since she had a nodular thyroid, it was difficult to say these were anything more than nodular degeneration of the thyroid, so I sent these nodules to the pathologists. In due course their report came through—they were all three parathyroid tumours. So her blood calcium came down again, she began to get signs of tetany which were not difficult to control and she got better again and she is still in Professor Perry's wards. We learn from this case that you may have more than one parathyroid tumour and this possibility must be remembered in the case under discussion this evening. I would like to ask what is a normal figure in the blood calcium and what should be regarded as abnormal? If we are looking for evidence of a tumour, what figure should we be suspicious of? I should have thought 11 was above the normal or even 10·6 mg. per cent.

*Dr. Lewis:* It averages about 10 but I think you would be on dangerous ground if you act on the assumption that 11 mg. per cent. is abnormal.

*Dr. Airth:* How would Mr. Cooke have diagnosed the finding on the X-ray pictures in this evening's case?

*Mr. Cooke:* In the absence of any other explanation, hyperparathyroidism is worth thinking about.

## Figures and Tables

**(a) f1:**
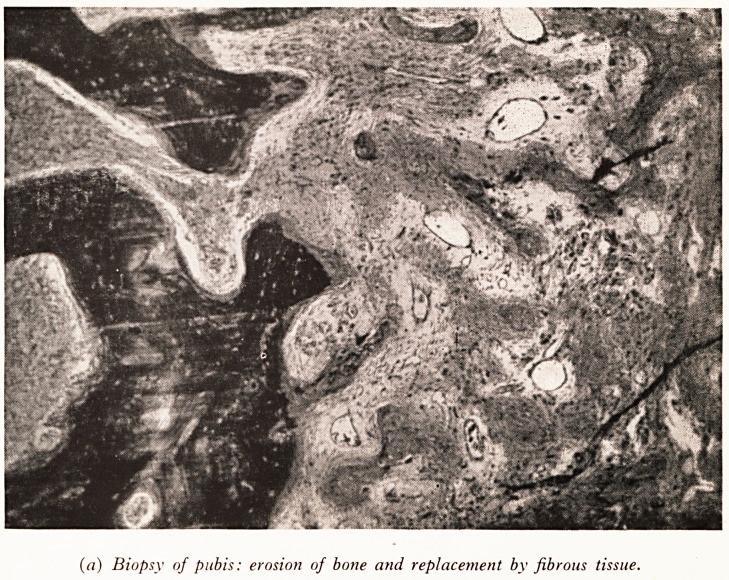


**(b) f2:**
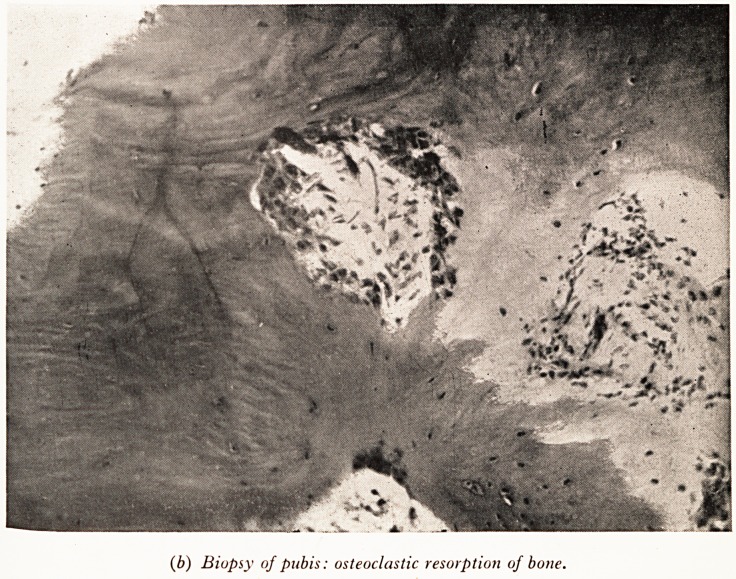


**(a) f3:**
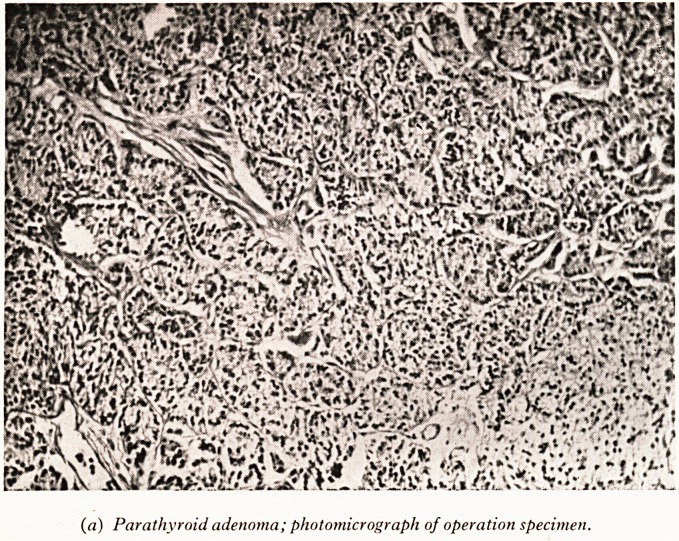


**(b) f4:**